# Design and analysis of a mobile robot with novel caster mechanism for high step-overcoming capability

**DOI:** 10.1038/s41598-024-63825-y

**Published:** 2024-06-14

**Authors:** Woojae Lee, Jeongeun Kim, Taewon Seo

**Affiliations:** 1 School of Mechanical Engineering, Hanyang University, Seoul, 04763 Republic of Korea; 2 HD Hyundai robotics, Robot Development Team, Seongnam, Gyeonggi 13553 Republic of Korea

**Keywords:** Engineering, Mechanical engineering

## Abstract

The mobile robot market is experiencing rapid growth, playing a pivotal role in various human-centric environments like restaurants, offices, hotels, hospitals, apartments, and factories. However, current differential-driven mobile robots, employing conventional casters and wheel motors, encounter limitations in surmounting uneven surfaces and high steps due to constraints caused by wheel and caster dimensions. While some robots address these challenges by incorporating optimized wheel shapes and additional motors, this invariably leads to an increase in both size and cost. This research introduces an innovative solution; a novel caster-wheel mechanism designed to enhance the high-step overcoming capability of mobile robots without necessitating alterations to their overall size and structure. By incorporating a sub-wheel linked to a passive joint, the driving force is effeciently converted into a vertical force, thereby empowering the mobile robot to navigate obstacles 85% larger than its caster-wheel radius. Crucially, this innovative caster can be seamlessly manufactured and integrated, offering the potential for widespread adoption as a replacement for conventional casters. Validation through comprehensive simulations and experiments conducted on a prototype robot has been presented in this article, demonstrating its effectiveness even at a robot velocity of 0.1 m/s. This pioneering solution holds significant promise for diverse applications across various mobile robot configurations, presenting a compelling avenue for further exploration and implementation in the field.

## Introduction

In recent years, the mobile robot market has experienced substantial growth, solidifying robots as integral components of human existence^1^. Furthermore, a significant percentage of newly established startups are venturing into the service robot market, contributing significantly to the overall landscape of robot companies in the United States. These statistics, alongside comparable data, underscore the necessity for formal advancements in the realm of service robots, emphasizing the importance of knowledge transfer and comprehensive literature reviews^2^. The surge in demand for mobile robots is primarily attributed to factors such as the aging population and the rise in remote work practices, accentuated by the recent COVID-19 pandemic. This context underscores the imperative for continued development and exploration in the field of service robots^3–6^.

A common mobile robot is typically composed of two active wheels and two passive wheels, with these wheels and casters serving as crucial elements in the robot’s functionality, particularly in environments with variable floor conditions. The challenge arises when mobile robots navigate confined spaces, demanding more exertion compared to wheeled robots^7^. In settings featuring substantial obstacles like stairs, the need for mobile robots capable of operating efficiently in restricted spaces becomes pronounced. This necessitates considerable advancements beyond the capabilities of conventional wheeled robots designed for free movement in confined areas^8,9^.

In exploring the caster-wheel mechanism aspect of the study, the mobility of mobile robots faces constraints in environments featuring elevated steps. The robot’s movement is limited to distances within the radius of the caster wheel, posing a challenge that necessitates substantial motor power and advanced technologies to overcome^10–12^. Various caster mechanisms have been extensively investigated to address this limitation. In Refs.^13^ and^14^, the authors devised a system to surmount differences in the front caster of a wheelchair by incorporating passive plates and locking devices, enabling the overcoming of high-step obstacles^15^. However, this solution is constrained by the requirement for additional motors^16^. The system’s configuration, with the assistant plate crossing the swivel radius, undergoes significant changes dependent on caster size, posing potential challenges in the overall robot system configuration. Another approach, as presented in Ref.^17^, involves the development of a unique shock-absorbing caster system for a compact design through the integration of a caster and suspension. While effective in traversing gaps and steps, the introduction of a suspension system in shock-absorbing casters increases their size, presenting difficulties in maintaining a consistent spring force with the coil suspension. In the case of this caster, it can be compared with the structure of the model ’Whegs’^18,19^. In the case of ’Whegs’, it rotates with the force of the motor based on the center of rotation, and the leg presses the upper surface of the ground to overcome the step difference. However, in the flat ground, since it is not in the form of a wheel, the robot vibrates. In the case of a novel caster, the main wheel has the effect of overcoming the step difference by contacting the vertical wall of the step and then the center of the main wheel contacts the step difference between the sub wheel and the main wheel. In addition, in the flat ground, a general cast increases the weight distribution and stability of the mobile robot in the form of a normal cast.

**Figure 1:**
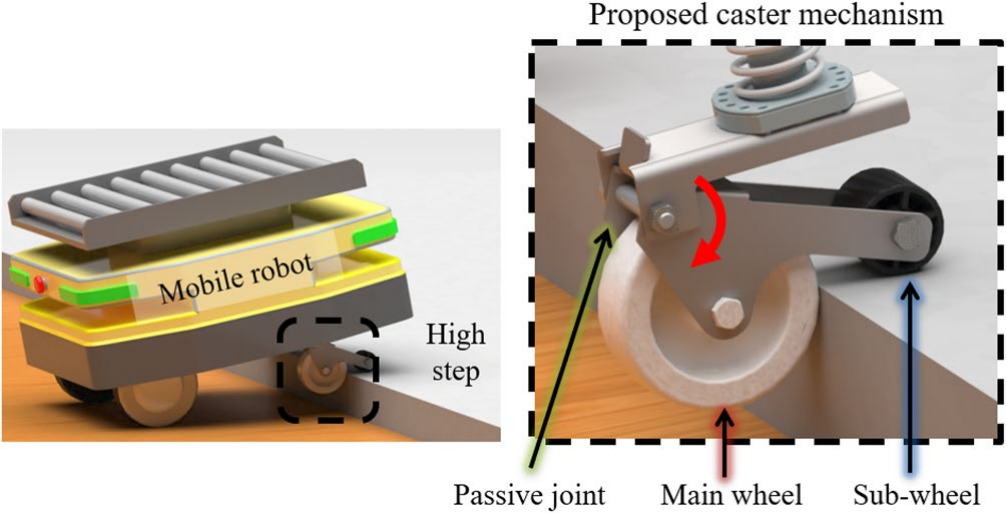
Proposed caster mechanism and overcoming high step of mobile robot with novel caster.

To address these challenges, this article introduces an innovative caster mechanism designed to surmount high steps. As depicted in Fig. 1, the mobile robot system encounters a high step, resulting in a horizontal reaction force on the caster wheel. Leveraging this reaction force, the sub-wheel connected to the passive joint descends in the direction of gravity, generating a vertical reaction force. This novel caster wheel system enables the robot to overcome steps exceeding 85% of the caster wheel radius. In the case of a general non-powered caster, it is difficult to overcome the step of more than 30% of the wheel diameter^14^. In the case of the novel caster, it is possible to overcome the step of more than the wheel diameter, and the performance of overcoming the step of more than 80% can be confirmed through experiments. It demonstrates a performance that is twice as effective as ordinary casters. Importantly, the proposed caster mechanism is meticulously designed to stay within the swivel radius of a typical caster system, eliminating the need for additional actuators. This design advantage facilitates enhanced step-overcoming capabilities by seamlessly replacing only the caster within the existing mobile robot system.

The subsequent sections of this paper is structured as follow. Section "Concept design" provides an overview of the conceptual design of the novel caster and delves into a scenario illustrating the capability of high step overcoming. In Sect. "Mechanical configuration", the caster mechanism and its kinematics is introduced, outlining the calculation of reaction forces and passive joint angles during high-step navigation. The distinct roles of each component of the novel caster are then explained. Moving forward to Sect. "Optimal design on the design parameters", optimization and validation processes through simulations are introduced, addressing various parameters. The assembly of the prototype and the corresponding experiments are detailed in Section "Prototype assembly". Section "Conclusions" serves as the conclusion, summarizing our findings, with a discussion on potential future avenues of this research.

## Concept design

**Figure 2:**
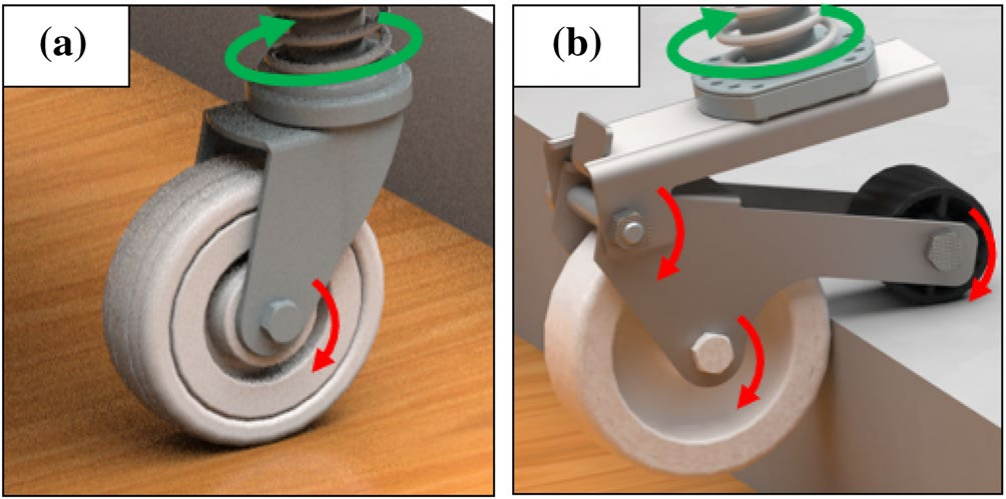
The red arrow indicates the rotation of the wheel and passive points. The green arrow indicates the gravity direction rotating point. (**a**) General caster design: It is largely divided into two parts. main wheel and gravity direction rotating joint and (**b**) Novel caster concept design: It is largely divided into four parts. main wheel, sub-wheel, passive joint, and gravity direction rotating joint.

This section explains the functionalities of caster units, beginning with a definition of the challenges associated with overcoming high steps using a conventional caster. A comparative analysis between the conventional and novel caster is also presented. Figure 2a shows the shape of a typical caster. It consists of a rotating wheel joint and a gravity-direction joint. There is a limitation in that the general caster wheel cannot overcome high steps. However, this issue can be addressed by increasing the size of the caster wheel. If the wheel size is increased, there is a limit to the increase in the system size of the entire mobile robot. The caster wheel size of typical mobile robot is approximately 50-75 mm. With such a caster, it is possible to overcome steps with heights of 16-25 mm. To solve the above problem, the main functions of the concept design that can overcome the limitation of the novel caster unit are described. We added a passive joint as shown in Fig. 1. In Fig. 2b, the novel caster adds a structure with a sub-wheel attached in the opposite direction to the main wheel of the caster. To overcome the high-step, the sub-wheel moves vertically using the reaction force generated by the main wheel. Using this vertical reaction force, the main wheel of the caster increases its ability to overcome the step.

### High step-overcoming scenario

**Figure 3:**
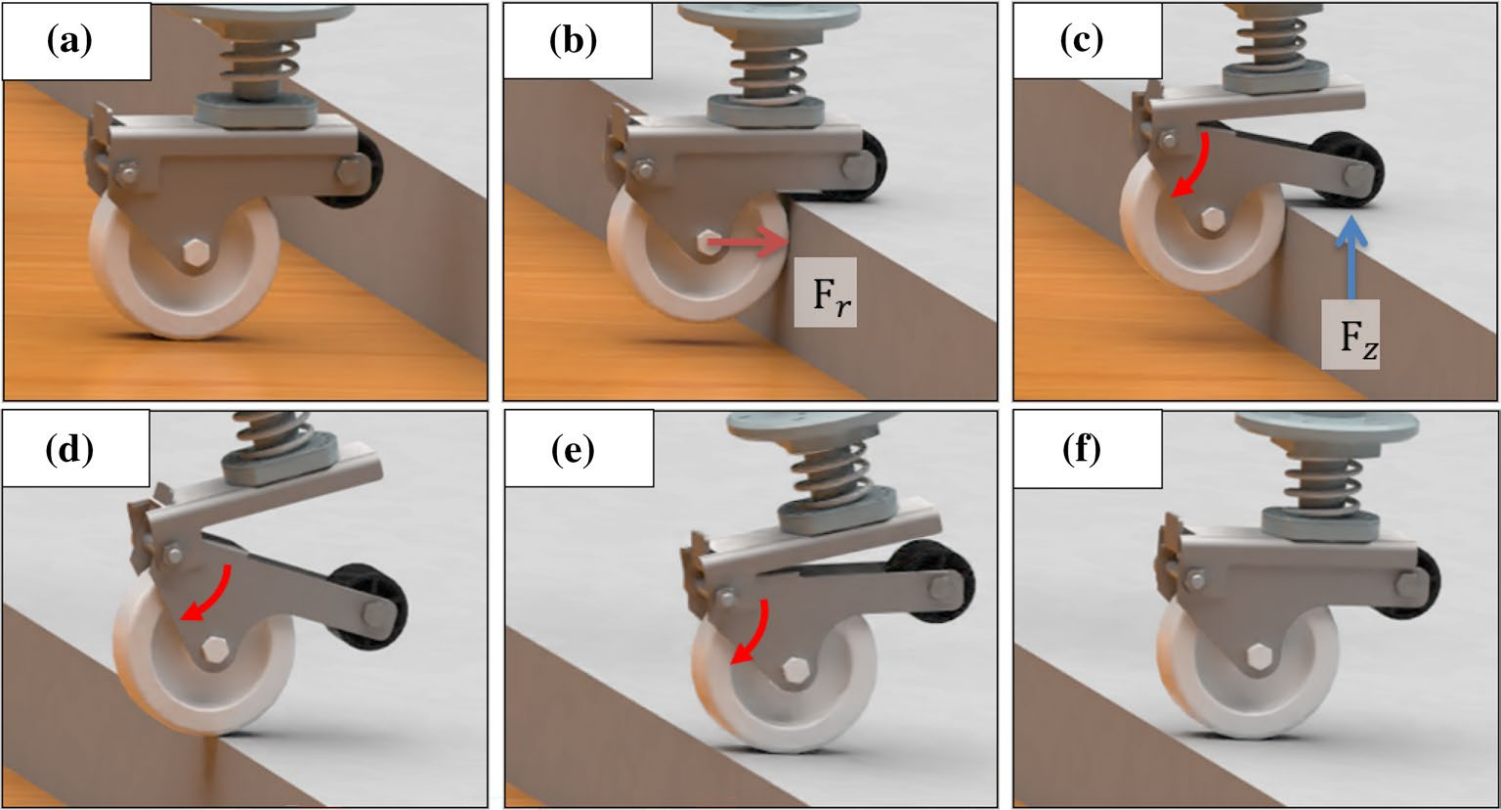
High step-overcoming scenarios for novel caster. (**a**) Novel caster unit when moving on flat surfaces. A force of $F_{r}$ occurs in the caster main wheel as much as the driving force of the mobile robot. (**b**) The step obstacle-faced moment. In process (**c**), $F_{r}$ is transformed to $F_{z}$, steps and reaction forces occur in the sub-wheel. (**d**) to (**f**) overcoming high step.

A novel caster is used to overcome this high step as shown in Fig. 3 to demonstrate its high step-overcoming ability. The robot overcomes the high step according to the following scenario: On flat ground, it operates in the same manner as a general caster as shown in Fig. 3a.

- When contacting a step similar to the center height of the sub-wheel, the sub-wheel contacts the step and then contacts the upper surface of the step. The caster main wheel contacts the high step as shown in Fig. 3b. If the height of the step is lower than the sub-wheel height, the main wheel contacts the wall surface of the step first. A force of $F_{r}$ occurs in the caster main wheel as much as the driving force of the mobile robot.
- In Fig. 3c, the sub-wheel connected to the passive joint descends in the vertical direction, producing a vertical reaction force at the step. As $F_{r}$ changes to $F_{z}$, reaction forces occurs in the sub-wheel.
- The main wheel is raised to a height that can overcome the step as shown in Fig. 3d.
- Figure 3e to f return to its original form.

The horizontal reaction force issued by the main caster wheel when it comes into contact with the high step lowers the sub-wheel connected to the passive joint in the vertical direction, lifting the caster in the vertical direction. Figure 3 represents a novel caster structure and an overcoming scenario. It is simple. However, this improved the mobile robot’s ability to overcome steps by more than two times.

## Mechanical configuration

**Figure 4:**
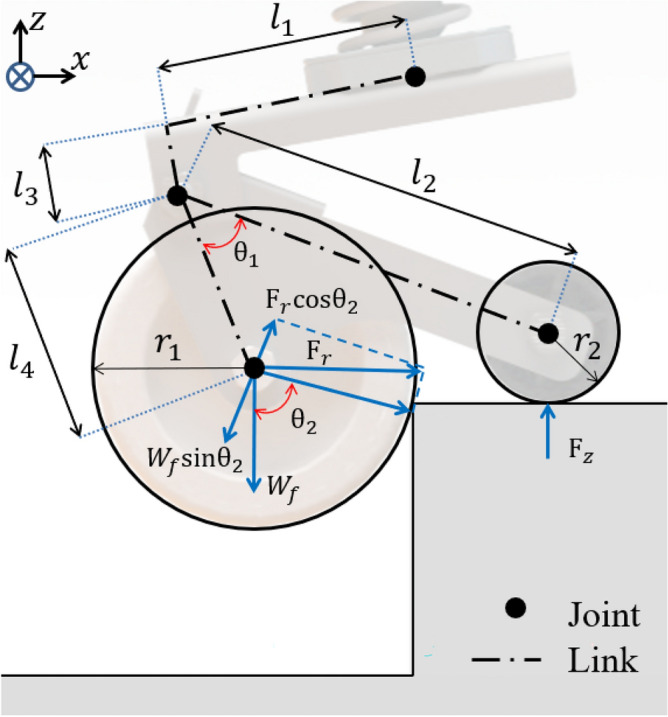
The schematic of the novel caster required the track length and key angle to determine the design conditions. $r_{1}$ and $r_{2}$ represent the radius of caster main and sub-wheel. $\theta _{1}$ is the composition angle of the caster. $\theta _{2}$ is the angle of the force generated by the contact of the caster main wheel. $l_{1}$ to $l_{4}$ are the part lengths. $W_{f}$ represents the vertical load applied from the main wheel.

All joints were configured as passive joints. The position of each axis is defined. The sub-wheel was defined as located in the driving direction of the mobile robot. It was defined to maintain a form in which only the main wheel of the caster came into contact with the ground when driving on flat land. Figure 4 describes the rotation axis as a variable in the design parameters.

1. $l_{1} < l_{2}$ : The center of the main wheel is located behind the Z axis of rotation, and the sub-wheel is always located in the driving direction of the mobile robot.
2. $l_{4}cos\theta _{1} < l_{2}$ : The vertical load point is located behind the main sub-wheel. It maintains the same shape as Fig. 4. In the case of $l_{4}cos\theta _{1} > l_{2}$, if the vertical load point acting on the caster is located between the sub-wheel and the main wheel, the main wheel and sub-wheel are in contact with the ground simultaneously.

### Kinematics

To overcome the high step, the caster’s main wheel falls off the floor when the sub-wheel contacts the ground on the step, as shown in Fig. 4. The mobile robot is driven by force ${F_r}$. It undergoes a horizontal reaction if the main wheel contacts the step. The sub-wheel contacts the ground as the reaction force rotates relative to the passive joint. ${F_r}$ changes into a vertical reaction force of ${F_z}$. This can be defined by Eq. (1)^20^.

1 $$\begin{aligned} F_{z} = \frac{l_{4}}{l_{2}}F_{r}cos\theta _{1} \end{aligned}$$

From Eqs. (2), (3), using the vertical reaction force of the sub-wheel, the center of the main wheel reaches the height of the step. $W_{f}$ represents the vertical load applied from the main wheel. $F_{r}$ is greater than the load $W_{f}tan\theta _{2}$ in the load direction of the mobile robot, and the main wheel can overcome this step. The main wheel can overcome the high this large step^21^.

2 $$\begin{aligned}{} & {} F_{r}cos\theta _{2} \ge W_{f} sin\theta _{2} \end{aligned}$$

3 $$\begin{aligned}{} & {} F_{r}\ge W_{f} tan\theta _{2} \end{aligned}$$

From Eq. (4), it is possible to define $F_{z}$ in which $F_{r}$ is generated in the sub-wheel^16^. The ability to overcome this step can be optimized through ${F_z}$ optimization of the sub-wheel. We can improve the step-overcoming ability of the novel caster by maximizing ${F_z}$. In the next chapter, ${F_z}$ optimization was performed through design variables and simulation processes.

4 $$\begin{aligned} F_{z}\ge \frac{l_{2}W_{f}tan\theta _{2}}{l_{4}F_{r}cos\theta _{1}} \end{aligned}$$

**Figure 5:**
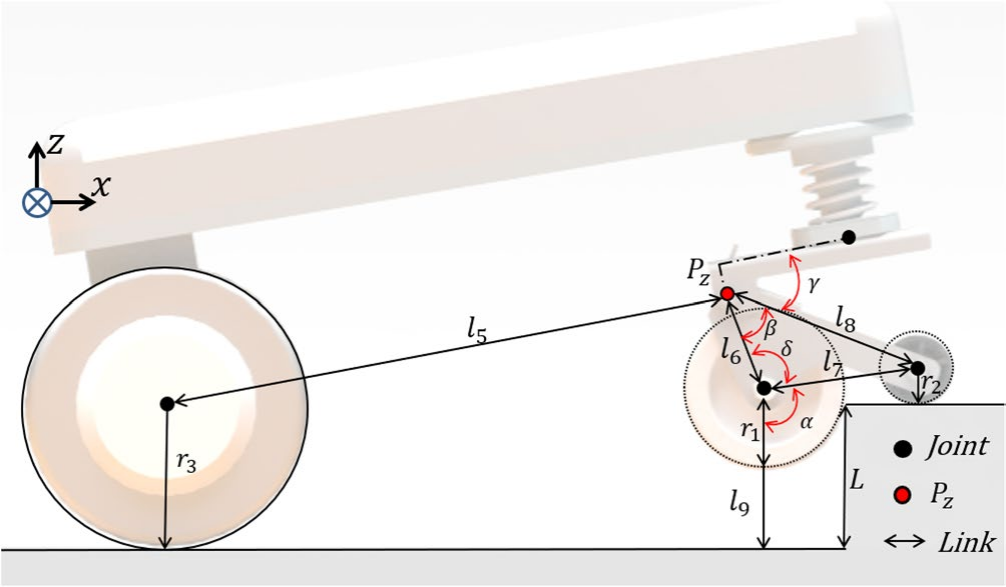
The schematic of the novel caster design parameters. $\alpha$, $\beta$, $\delta$ are the angles for each joint that occurs when overcoming a high step. $\gamma$ needs to optimize a variable. $l_{5}$ to $l_{9}$ are part length when overcoming the high step, $P_{z}$ represents the height of the passive joint from the flat surface, and *L* is the step height.

**Table 1:** Design parameters list.

Design parameter	Unit	Lower bound	Initial value	Upper bound
$l_{5}$	mm	–	305.2	–
$l_{6}$	mm	49.6	50	56.3
$l_{7}$	mm	53.3	69.6	76.1
$l_{8}$	mm	80.5	93	105.1
$l_{9}$	mm	–	65	–
$r_{1}$	mm	–	37.5	–
$r_{2}$	mm	–	16	–
$r_{3}$	mm	–	65	–
*L*	mm	–	65	–

## Optimal design on the design parameters

In Fig. 5, we optimize for the design variable parameters $l_{6}$, $l_{7}$, and $l_{8}$. The height of the step to be overcome is defined by the radius of the main-wheel caster and the center coordinate of the sub-wheel. The radius of the caster main wheel and sub-wheel were fixed at 65 and 16 mm, respectively. The optimization of each coordinate according to each constraint is required.

The angles of the passive point and ${F_z}$ were minimized through parameter optimization. In Fig. 5 shows that the length of $l_{5}$ is determined by the length of the driving part of the mobile robot. As the length of the drive part increases, the passive joint angle decreases. However, increasing the length of $l_{5}$ results in a larger overall robot size. Hence, $l_{5}$ is fixed at 305.2 mm. The levels for each coordinate of the caster are determined based on the constraints listed in Table 1. The Z-coordinate of the sub-wheel depend on its radius. To preserve its original shape, the sub-wheel must remain in contact with the main body of the caster. Therefore, the radius of the sub-wheel is set to a fixed value of 26 mm.

### Design parameter optimization

Because the derived mathematical model in this optimization study is a multi variable with non linear constraints, a MATLAB function called *gamultiobj* was used to optimize the constructed model. This is a gradient-based function that can be used to search for and find all possible local minima that satisfy the given objectives^22,23^. The maximum overcoming, which is the potential step height from the ground to the center of the sub-wheel, was set at 65 mm. At a height of 65 mm or higher, the sub-wheel collides with the step in the vertical direction, making it impossible to overcome the step. The angle $\gamma$ was optimized when the center height of the caster’s main wheel was above the height of the step through equal weight distribution. From Eq. (5), $\alpha$, $\beta$, and $\delta$ can be determined using trigonometric methods based on the range as well as the minimum and maximum values of the lengths of $l_{6}$, $l_{7}$ and $l_{8}$. $P_{z}$, the height of the passive joint, was calculated according to the maximum height $l_{6}$ in Eq. (6). According to Eq. (7), $\gamma$ can be obtained based on the length of $l_{5}$. When the initial value is reached, it can be calculated based on the value to be changed. Equation (8) represents $F_{z}$ generated in the sub-wheel when overcoming the step indicated in Fig. 4. By minimizing $F_{z}$, it is possible to minimize the driving force required to overcome the step.

**Figure 6:**
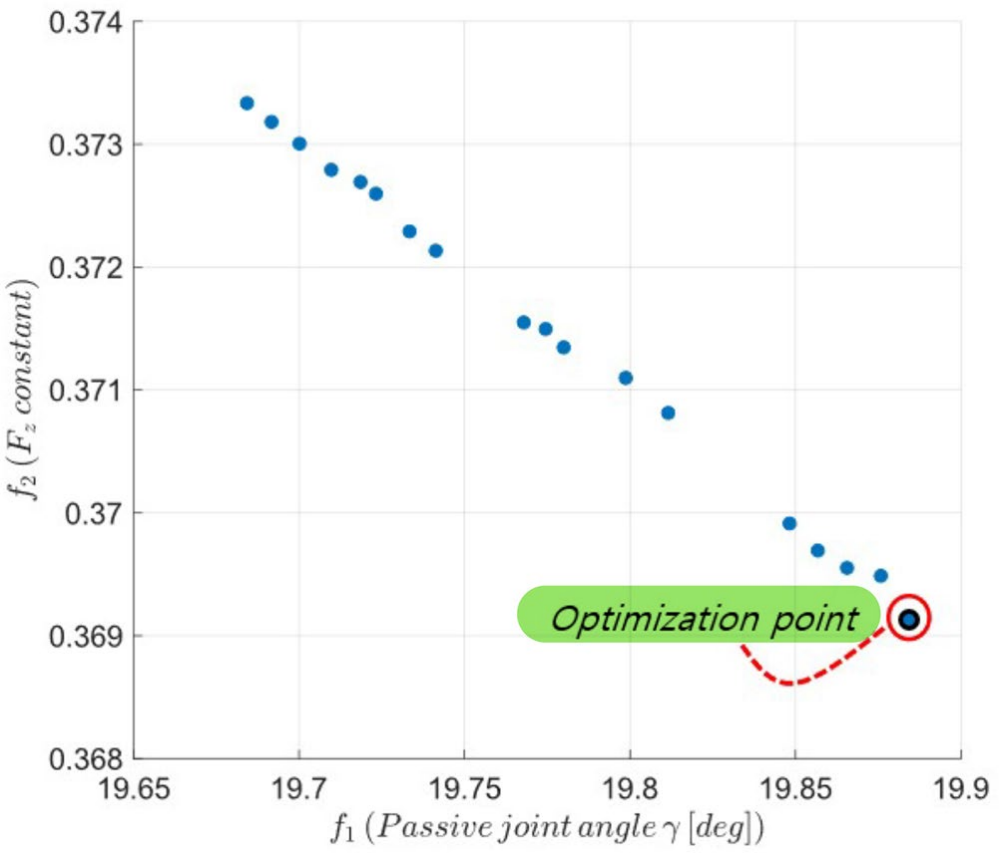
Design parameter result graph, $f_{1}$ represents the passive point angle $\gamma$ [deg], $f_{2}$ represents the $F_{z}$ constant value. $f_{1}$ and $f_{2}$ represent an inverse relationship with each other. The red circle represents the optimum point of $f_{1}$, $f_{2}$.

5 $$\begin{aligned} \alpha= & {} sin_{}^{-1}\left( \frac{r_{2}+L-l_{9}}{l_{7}} \right) ,\,\beta =cos_{}^{-1}\left( \frac{l_{6}^{2}+l_{8}^{2}-l_{7}^{2}}{2l_{6}l_{8}} \right) ,\, \delta =cos_{}^{-1}\left( \frac{l_{6}^{2}+l_{7}^{2}-l_{8}^{2}}{2l_{6}l_{7}} \right) \qquad when \; r_{2} + L < l_{9} \end{aligned}$$

6 $$\begin{aligned} P_{z}= & {} L + l_{6}cos(\alpha -90\,^{\circ }) \end{aligned}$$

7 $$\begin{aligned} f_1= & {} \gamma = cos^{-1}\left( \frac{P_{z} - r_{3}}{l_{5}} \right) - \beta \end{aligned}$$

8 $$\begin{aligned} f_2= & {} F_{z} = \frac{l_{8}W_{f}tan\theta _{2}}{l_{6}F_{r}cos\theta _{1}} \end{aligned}$$

By optimization, $l_{6}$ = 49.8 mm, $l_{7}$ = 73.9 mm, and $l_{8}$ = 105.4 mm. As shown in Fig. 6, $f_{1}$ (passive joint angle) and $f_{2}$ ($F_{z}$ constant) were optimized. The Y-axis represents $f_{2}$ ($F_{z}$ is a constant), whereas the X-axis represents $f_{1}$ (passive point degree). As the passive joint angle increases, the value of the $F_{z}$ constant decreases inversely. $f_{1}$ = 19.87$\,^{\circ }$ and $f_{2}$ = 0.369 were selected. The reaction force and angle values are verified through simulations, as described in the following section. By minimizing the force of $F_{z}$, it is possible to minimize the required $F_{r}$. That is, it is possible to overcome the step with the minimum driving force of the mobile robot.

**Figure 7:**
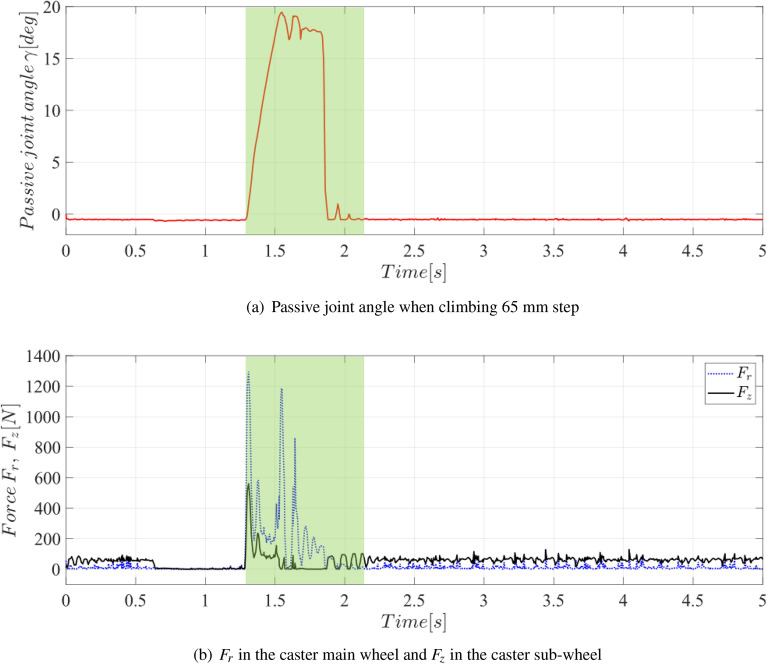
Novel caster dynamic simulation result when overcoming 65 mm step of novel caster. The green area is the climbing section. (**a**) Passive joint angle when climbing 65 mm step. (**b**) F_r_ in the caster main wheel and F_z_ in the caster sub-wheel.

### Simulation

**Figure 8:**
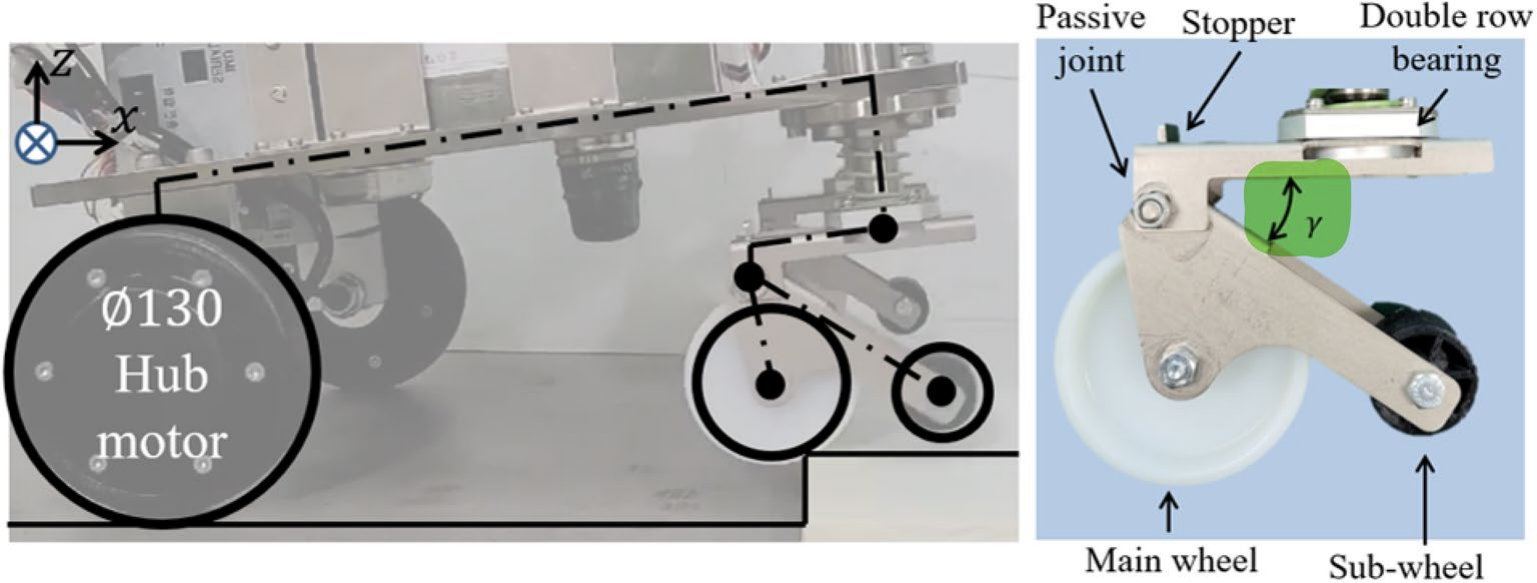
Novel caster implementing a similar form to the regular caster using the stopper and double row bearing. Power was implemented using a $\phi$130 mm hub motor.

The simulation of the novel caster was conducted based on design parameter optimization values from previous section. We conducted a step-overcoming simulation using Altair’s motioview dynamics engine for verification. A driving speed of 0.1 m/s was applied to the mobile robot system. The passive joint angle is set to a maximum value of $19.87\,^{\circ }$. The maximum step overcome was 65 mm. Figure 7a presents the novel caster passive joint angle change of $f_{1}$ (passive joint angle) when overcoming a step. Figure 7b of the simulation results shows the horizontal reaction force generated by the main wheel of the caster when a high step collides. The $F_{r}$ result value was confirmed. In Fig. 8b, the caster main wheel represents $F_{z}$, which occurs when the high step collides, as the sub-wheel descends in the vertical direction owing to the horizontal reaction force generated when the high step collides. $F_{z}$ was 0.369 times that of $F_{r}$. A vibration reduction effect in the Z-direction of the main body of the mobile robot was observed by minimizing the angle change. Moreover $F_{z}$ minimizes the hub motor capacity. The simulation results confirmed the ability of novel caster to overcome the step. In addition, the necessary motor torque and components can be selected according to the speed.

## Prototype assembly

Base on the previous simulation results and the design parameter optimization process, we proceeded with the manufacture of the components and prototypes of each composition. The specifications of the rotary double-bearing and joint pin were selected as shown in the figure. The stopper was designed at an angle through optimization. In order to implement the angle of $f_1$ selected in Fig. 6, the angle that can rotate to the maximum based on the passive point in the circular shape was implemented through the stopper. Figure 8 shows the shape when the caster shape is the maximum rotated angle. Figure 8 illustrates the manufactured prototype. A stopper was designed to optimize the angle. It is possible to drive up to a selected maximum speed of 0.1 m/s through the simulation results. The entire mobile robot system shown in Fig. 8 consists of two $\phi$130 mm hub motors and two novel casters. It consists of a 24 V battery, a main controller, and a 200 W motor driver. Each joint was constructed as a rotating joint using a hinge pin.

## Experiments

An experiment was conducted to verify the feasibility of novel caster. The step height was set by lowering it by 5 mm from the 65 mm step standard, which is the maximum achievable height with the currently designed caster. The robot speed was fixed at 0.1 m/s and the test was then conducted. In the case of the novel caster, power was generated from the back. Therefore, it is possible to verify the ability of the novel caster to overcome the steps. In the case of an overall mobile robot, it is necessary for the caster to overcome the step and check the step capability using a $\phi$130 mm hub motor.

### Novel caster

**Figure 9:**
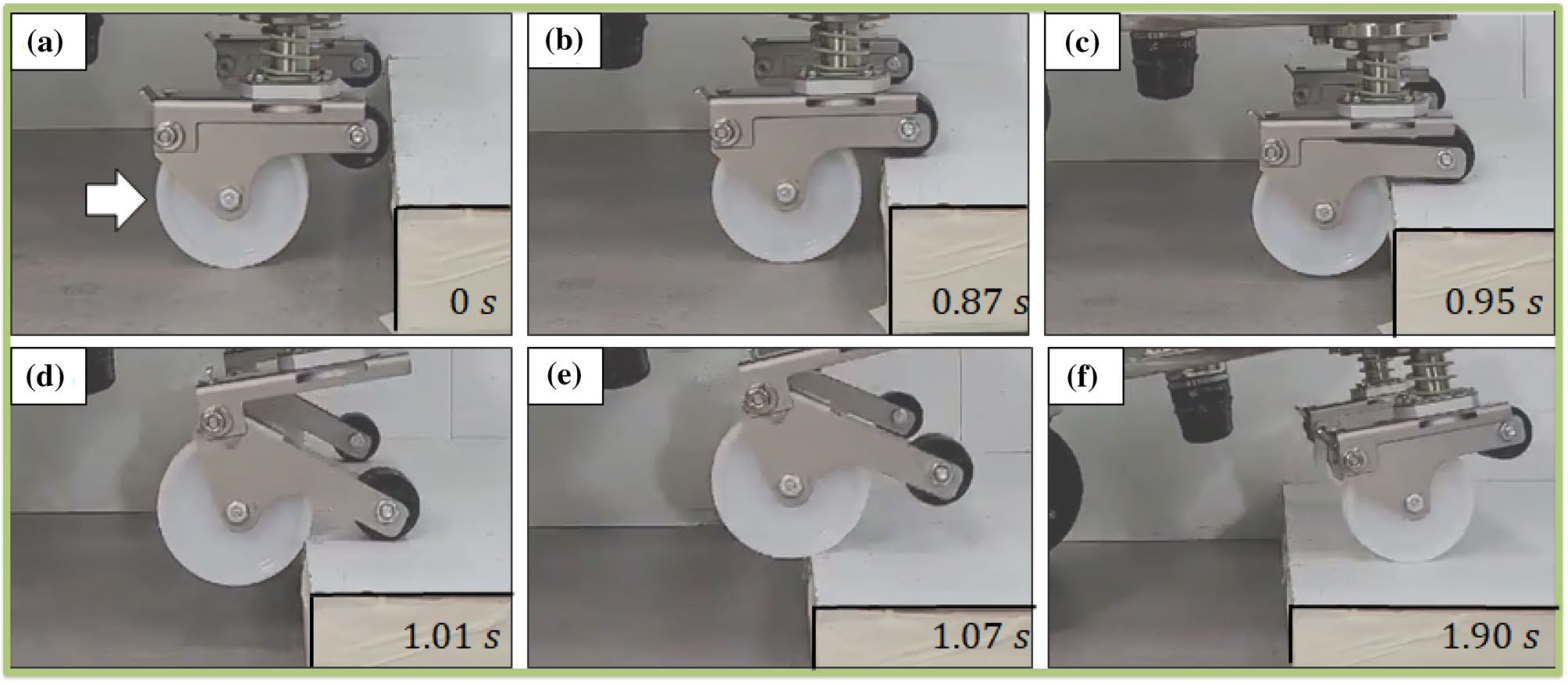
Novel caster step obstacle climbing snapshot; 65 mm step overcoming capability test of the novel caster. The picture shows the order and the time sequence. The white arrow indicates the direction of the robot’s progress.

The first experiment verified the ability of the novel caster to improve the step-overcoming performace. Figure 9 shows a snapshot of the overcoming scene of the novel caster. In Fig. 9b, the step is first contacted by the sub-wheel. In Fig. 9c, a force of $F_{r}$ is generated on the caster main wheel. In Fig. 9d and e, $F_{r}$ is changed to $F_{z}$ and the caster’s main wheel overcomes the high step. Returning to its original form as shown in Fig. 9f.

### Overall mobile robot

**Figure 10:**
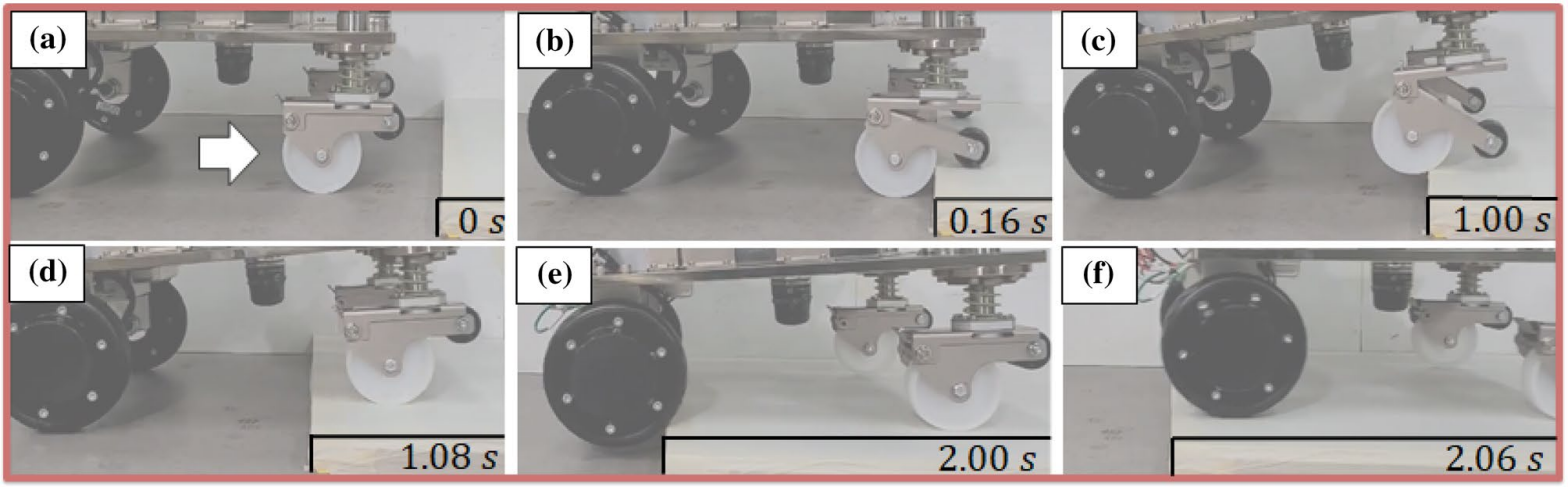
Mobile robot with the novel caster step obstacle climbing snapshot; 30 mm step overcoming capability test of overall system. The picture shows the order and the time sequence. The white arrow indicates the direction of the robot’s progress.

The second situation verifies the overall ability of the mobile robot to overcome the step performance. In the case of this experiment, the experiment was conducted at a lower height than the high step-overcoming scenario. The sub-wheel is not in contact with the upper surface of the step first, and the main wheel is in contact with the wall surface of the step. Figure 10 illustrates a snapshot of the overcoming scene of the mobile robot. In Fig. 10b, the step is first contacted by the sub-wheel. In Fig. 10c and d, the caster main wheel overcame this step. The mobile robot overcomes the steps shown in Fig.  10e and f. Sufficient frictional force is required for the novel caster and driving wheel to overcome this step. Because the power is located on the rear wheel of the mobile robot, it is possible to overcome only the lower height compared with the novel caster. However, in the case of the rear wheel, the step must be overcome only by the driving force of the wheel. In the case of a typical active wheel, it is difficult to overcome the step of more than 30% of the diameter^24^. Since there is no driving force of the novel caster, the step difference must be overcome only by the near wheel driving force. Therefore, it is difficult to overcome the high step (Supplementary Video 1).

### Experimental results

The experimental results, confirmed that the novel caster could overcome up to 65 mm, similar to the simulation results. However, in the case of the overall mobile system, it was possible to overcome the 30 mm step. Two problems were identified in this study. Problems with the grip force of the hub motor may occur depending on the center of mass location. If the hub motor does not generate sufficient grip force, it can result in the motor spinning without effective movement. In such cases, the insufficient ability to overcome can be attributed to the frictional force of the motor. Therefore, it is crucial to reassess the overall center of mass of the mobile robot to achieve optimal performance. In results, through the optimization of the design parameters $f_1$ and $f_2$, it is possible to overcome the difference more than the wheel diameter in the case of the novel caster, and the overcoming performance of the difference of 80% or more was confirmed through experiments. If the center of the sub-wheel is lower than the height of the step, the sub-wheel is in contact with the wall surface, not the upper surface of the step. As the sub-wheel moves to the floor based on the passive point, it is impossible to play the role of the novel coaster. Within the size of the system in which the caster can be implemented, it is possible to overcome the step with the minimum driving force and angle by utilizing the optimal values of $f_1$ and $f_2$ at a step lower than the center of the sub-wheel.

## Conclusions

This study proposed a novel mobile robot caster to improve the step-overcoming ability of mobile robots. By utilizing a sub-wheel linked to a passive joint, the driving force is converted a vertical force. This augments the mobile robot’s capacity to surmount large steps. The design parameters applicable to mobile robots were analyzed and optimized to improve the step-overcoming capability. We verified this in simulations and real-world experimental environments, where mobile robots with new casters were investigated. Base on the simulations and experiments, a caster with a radius of 37.5 mm could overcome a step of 65 mm. In the experimental mobile robot system, the $\phi$130 mm hub motor could overcome a high step of 30 mm. In the case of the entire system, there was a limit to the ability of the caster to overcome the step using only the hub motor. Sufficient frictional force are required for the novel caster and driving wheel to overcome the step. Active wheels are located at the rear wheel of the mobile robot. It is only possible to overcome a lower height than that of the novel caster. In the near future, we plan to optimize the step-overcoming capability of the overall mobile robot system by changing the passive wheel of the caster to a hub motor.

## Supplementary Information

Supplementary Video 1.

## Data Availability

All data generated or analysed during this study are included in this published article.
